# Anemia ‐ an initial manifestation of Bing‐Neel syndrome: A case report

**DOI:** 10.1097/MD.0000000000031239

**Published:** 2022-11-18

**Authors:** Xiaoqian Yang, Zonglei Chong, Congcong Ma, Guifang Wang, Chunxia Yan

**Affiliations:** a Department of Neurology, Liaocheng People’s Hospital, Shandong, China; b Department of Neurosurgery, Liaocheng People’ s Hospital, Shandong, China; c Department of Hematology, Liaocheng People’s Hospital, Shandong, China.

**Keywords:** anemia, Bing‐Neel syndrome, case report

## Abstract

It is very likely that we will miss Bing-Neel syndrome (BNS) when its initial sign is anemia.

Patient concerns: A 59-year-old woman presented with episodic loss of consciousness, anemia, and extremity muscle strength scores (5-) and extremity tendon reflexes (++).

**Diagnoses:**

Magnetic Resonance Imaging (MRI) showed abnormal signal in the left hippocampus, left insula, and right occipital lobe. Quantitative serum immunoglobulins showed elevated immunoglobulinm (IgM) (60.6g/L). Bone marrow biopsy showed lymphoplasmacytic lymphoma (LPL) and tested positive for the MYD88 L265P mutation suggesting Waldenström macroglobulinemia (WM).

**Interventions:**

The patient underwent 3 plasma exchange treatments in the department of hematology followed by chemotherapy (cyclophosphamide for injection, bortezomib for injection).

**Outcomes:**

The patient’s condition improved after treatment.

**Lessons:**

Clinicians must remain vigilant, as BNS may be the only sign of WM progression in a patient well-controlled on treatment.

## 1. Introduction

Primary Waldenström macroglobulinemia (WM) is a rare disease of unknown etiology, with an annual incidence of about 3/1 million.^[1]^ Hyperviscosity manifests as cerebral infarction caused by monoclonal immunoglobulinm (IgM) protein in the blood.^[2]^

Clinical manifestations: Patients may develop symptoms associated with infiltration of hematopoietic or other tissues (such as anemia, lymphadenopathy, hepatosplenomegaly), or symptoms associated with monoclonal IgM protein in the blood (such as hyperviscosity, peripheral neuropathy).^[3]^ Hyperviscosity syndrome: up to 30% of patients experience symptoms related to hyperviscosity, resulting in blurred or lost vision, headache, dizziness, nystagmus, tinnitus, sudden deafness, diplopia, or often neurological deficits, ataxia and other systemic diseases. Peripheral neuropathy: about 20% of patients may have symptoms of peripheral nerve damage at the time of diagnosis.^[4]^ The most common neurological abnormalities are distal, symmetrically distributed, slowly progressive sensory or motor abnormalities manifesting as paresthesias or limb weakness. Cranial nerve palsy may occur in some patients. Abnormal ophthalmoscopy findings: thirty-four percent of patients had abnormal ophthalmoscopy results, which showed segmental twisting and dilation of retinal veins in a “sausage chain” pattern. Some patients may have retinal hemorrhages, exudates, and papilledema. Central retinal vein thrombosis may occur. Cryoglobulinemia: manifested as Raynaud’s phenomenon, urticaria, purpura, acrocyanosis or tissue necrosis. Gastrointestinal symptoms: rarely, monoclonal IgM protein may form extracellular amorphous material and deposit in the gastrointestinal tract lamina propria, causing severe malabsorption with diarrhea and steatorrhea. Others: other clinical manifestations are uncommon, such as osteolytic lesions, lung involvement, and skin lesions.

Notably, there are no signs or symptoms characteristic of WM.^[5]^ However, the presence of particular clinical findings can help to direct additional evaluation.^[6]^ In addition, we should further investigate any signs, symptoms, or other causes of laboratory or imaging findings to determine their likelihood of being associated with WM.^[7]^ A careful and systematic history taking can provide information not only on the presence of constitutional symptoms such as fevers, night sweats, or unintentional weight loss, but also for potential alternative causes for these symptoms.^[8]^ Symptoms associated with anemia are common in patients with WM, and include fatigue, malaise, and shortness of breath.^[9]^ Symptomatic hyperviscosity can induce recurrent episodes of spontaneous epistaxis, new-onset headaches, and blurred vision.^[10]^ In this report, we describe a case of anemia as the initial manifestation of Bing‐Neel syndrome (BNS).

## 2. Case presentation

**Figure 1.**
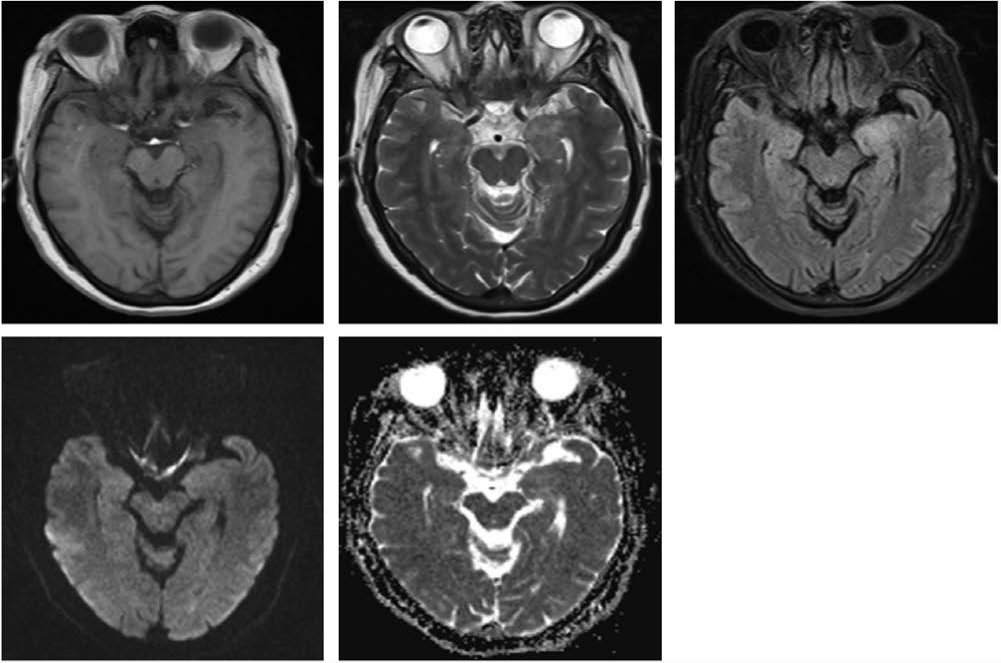
The left hippocampus showed slightly longer T1 and T2 signal plaques, slightly higher signal on FLAIR sequence, no diffusion limitation on DWI, slightly higher signal on ADC. ADC = apparent diffusion coefficient, DWI = diffusion-weighted imaging, FLAIR = fluid attenuated inversion recovery, T1 = T1-weighted imaging, T2 = T2-weighted imaging.

**Figure 2.**
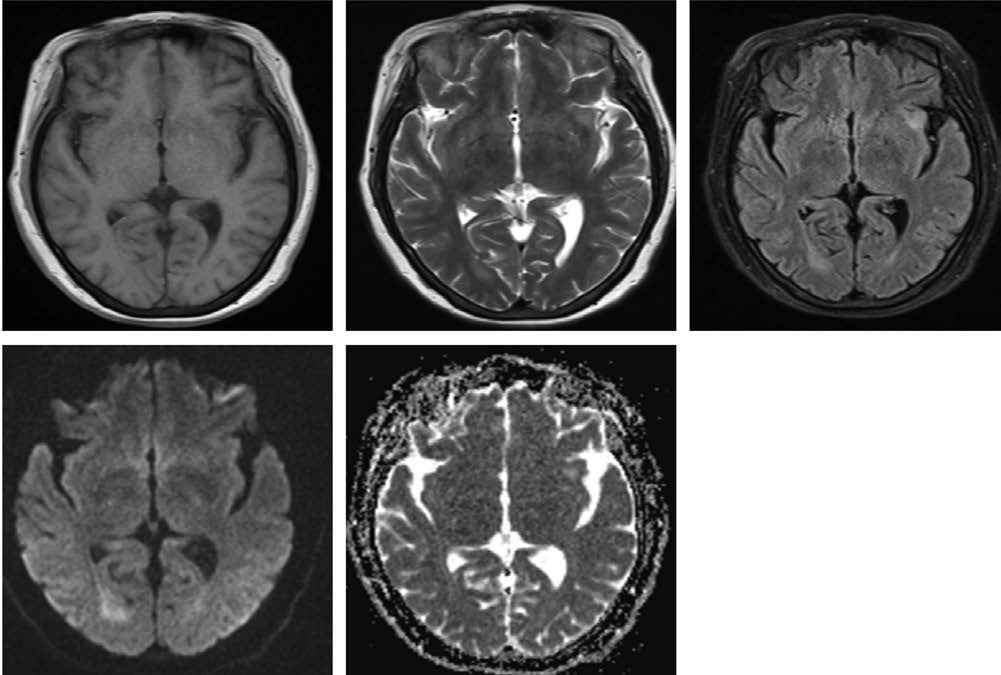
The right occipital lobe and left insular lobe showed patchy slightly longer T1 and slightly longer T2 signals, slightly high signal on FLAIR sequence, limited diffusion on DWI, and high signal on ADC. ADC = apparent diffusion coefficient, DWI = diffusion-weighted imaging, FLAIR = fluid attenuated inversion recovery, T1 = T1-weighted imaging, T2 = T2-weighted imaging.

**Figure 3.**
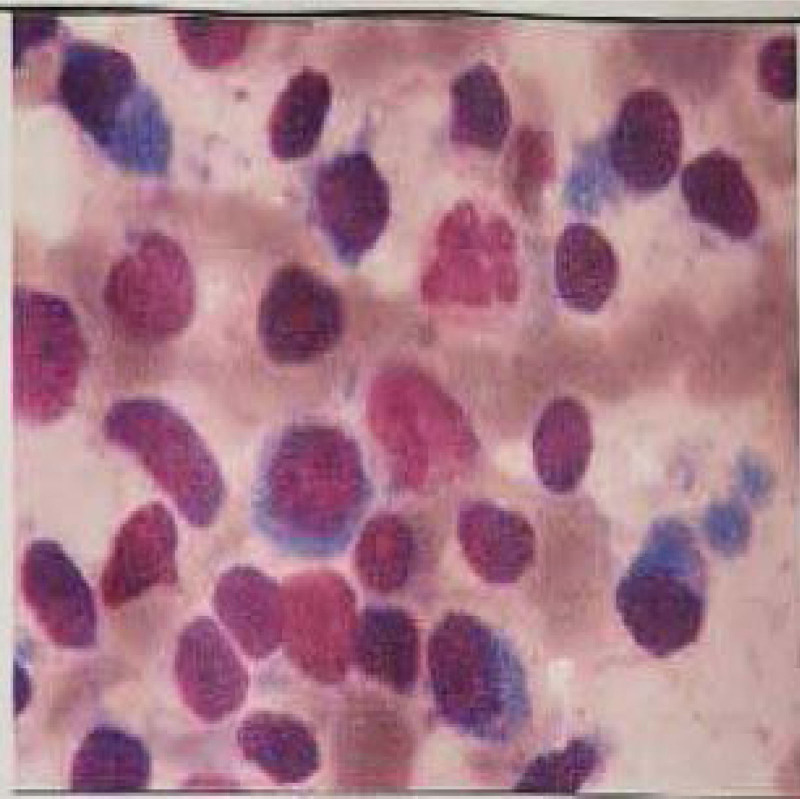
Bone marrow aspiration: we can see hyperactive myeloid hyperplasia, abnormally increased lymphocyte ratio, easy to see plasmacytoid lymphocytes.

In May 2020, a 59-year-old female came to Liaocheng Brain Hospital for treatment with episodic loss of consciousness for 2 months, aggravating for 10 hours. Physical examination revealed the complexion, lips, palpebral conjunctiva are pale; poor mental status, extremity muscle strength grade (5-) and extremity tendon reflexes (++). The patient has a history of diabetes for three years, treated with metformin, and has good blood sugar control; hyperlipidemia for many years, treated with simvastatin; a history of myocardial infarction, sinus tachycardia, moderate anemia for one and a half years, and intermittent gingival bleeding; history of cerebral infarction for one year, no sequelae. After blood draw, carry out experimental examination and analysis: the patient’s blood analysis showed red blood cells 2.64 × 10^12^/L↓, hemoglobin 79 g/L↓, hematocrit 24.2%; liver biochemical examination showed total protein 104 g/L↑, albumin 30 g/L↓, globulin 74 g/L↑, leukoglobulin ratio 0.41↓; coagulation mechanism examination showed prothrombin time 13.8s↑, and the myocardial enzyme spectrum troponin I test result was 0.205 ng/mL↑. Brain Magnetic Resonance Imaging (MRI) and MRA showed weak local blood flow signal in the P4 segment of the left posterior cerebral artery; Abnormal signals in the left hippocampus, left insula, and right occipital lobe; multiple lacunar cerebral infarction (Figs. 1 and 2). We carried out the bone marrow biopsy and the morphological analysis of the bone marrow cells with Rigg’s staining, and the specimen showed abnormal B lymphocytes and abnormal plasma cells (Fig. 3). Flow cytometry was used to detect the antigens expressed on the cells, which showed that the abnormal plasma cells expressed CD45, CD38, CD138, cKaPPa, CD19; abnormal B cells express CD19, CD20, CD79b, CD1d, cKappa, but not CD11C, CD103, CD10, CD5, CD56, CD43, FMC7, sIgM, cLamda, CD138. Quantitative detection of MYD88 gene L265P mutation by real-time fluorescent polymerase chain reaction technology found that the samples were positive for MYD88 L265P mutation. Serum immunofixation electrophoresis confirmed elevated IgM monoclonal paraprotein, confirming the diagnosis of WM.

The patient underwent 3 plasma exchange treatments in the department of hematology followed by chemotherapy (cyclophosphamide for injection, bortezomib for injection), and the patient’s condition improved after treatment.

## 3. Discussion and conclusions

Primary WM refers to lymphoplasmacytic lymphoma (LPL) in the bone marrow with IgM monoclonal gammopathy in the blood.^[11]^ It is a rare disease with various onset forms, including anemia, lymphadenopathy, hepatosplenomegaly, transient ischemic attack (TIA), cerebral infarction, cerebral hemorrhage, peripheral neuropathy, and myocardial infarction.^[12]^ Anemia is the most common condition for which WM patients begin to seek treatment.^[13]^ Anemia in patients with WM may be due to bone marrow replacement by abnormal lymphocytes and plasma cells, iron deficiency, or hemolysis.^[14]^ We should do iron studies (that is iron, total iron-binding capacity and ferritin).^[15]^ We should examine gastrointestinal blood loss by upper, lower, and capsule (small bowel) endoscopy in patients with absolute iron deficiency (low iron saturation and low ferritin levels).^[16]^ In some patients, there may be evidence of functional iron deficiency (low iron saturation and normal or high ferritin levels).^[17]^ WM cells can produce hepcidin, which is a regulator of serum iron content, inducing a mixture of absolute and functional iron deficiency.^[18]^ In these cases, intravenous iron supplementation can improve the anemia and delay WM directed treatment.^[19]^ Rarely, anemia can be secondary to hemolysis. We should perform a hemolytic panel, including reticulocyte counts, lactate dehydrogenase, haptoglobin, direct Coombs test, and cold agglutinins in these patients.^[20]^ We should initiate plasma exchange immediately in patients with severe cold agglutinemia to remove cold agglutinin.^[21]^ We should rule out other causes of anemia, such as cobalamin and folate deficiencies; renal, hepatic, or thyroid dysfunction; nonautoimmune hemolysis (for example, hemoglobinopathies); or other primary bone marrow disorders.

The damage of primary WM to the central nervous system (CNS) mainly includes two reasons.^[22]^ Hyperviscosity syndrome: a series of clinical manifestations related to cerebral infarction caused by monoclonal IgM protein in the blood.^[23]^ In rare cases, marked hyperviscosity can lead to loss of consciousness, dementia, disturbance of consciousness, cerebral infarction, and cerebral hemorrhage.^[24]^ BNS: a syndrome caused by infiltration of the CNS by small lymphocytes and plasma cells.^[25]^ When small lymphocytes and plasma cells infiltrate the CNS, known as BNS, biopsy of the brain or meninges to demonstrate LPL infiltration is the gold standard for diagnosing BNS.^[26]^

According to MRI imaging findings, researchers distinguish two forms of BNS: the diffuse form presenting as meningeal and perivascular involvement and the tumoural form presenting as expansive lesions.^[27]^ The diffuse form: lymphoplasmacytic cells infiltrate the meninges or paravascular areas, and the blood-brain barrier is disrupted, manifesting as vasculitic edema.^[28]^ MRI shows long T1-weighted imaging (T1) and long T2-weighted imaging (T2) signals, diffusion weighted imaging (DWI) can be hypointense, isointense or mild hyperintensity, Apparent diffusion coefficient (ADC) is hyperintensity or normal signal, or enhanced signal.^[29]^ The tumoural form: Lymphoplasmacytic cells penetrate deep into the subcortical brain tissue and can be single or multiple masses.^[30]^ MRI often shows edema at the lesion site, showing long T2 signal and fluid attenuated inversion recovery (FLAIR) high signal.^[31]^

Diagnostic criteria of WM: serum immunofixation electrophoresis confirmed the presence of IgM monoclonal paraprotein; the proportion of small lymphocytes in bone marrow biopsy specimens was greater than or equal to 10%; 3.90% of patients had MYD88 L265P gene mutation.^[32]^

Treatment should start only for symptomatic WM patients. Plasmapheresis is still an important treatment for certain WM related complications with rapid but only temporary results. Treatment of WM includes rituximab-based combinations (with cyclophosphamide, bendamustine, fludarabine, chlorambucil, or proteasome inhibitors such as bortezomib, carfilzomib, ixazomib), and, less commonly, rituximab monotherapy. New targeted therapies include Bruton’s tyrosine kinase inhibitors (Ibrutinib, acalabrutinib, zanabrutinib), B-cell lymphoma 2-targeting agents (Venetoclax) and in selected cases may also include stem cell transplantation. We can select the treatments available today based on the patient’s age, presence of comorbidities, viscosity-related symptoms and the need for rapid cytoreductive surgery, and treatment-specific side effects.

Here, we present a case of anemia as an initial sign of BNS. A 59-year-old female presented with episodic loss of consciousness, anemia appearance, muscle strength grade 5-, and the tendon reflexes of the extremities were (++). MRI showed abnormal signal in the left hippocampus, left insula, and right occipital lobe. These evidences support the MRI findings of aggressive BNS but not the imaging findings of cerebral infarction due to hyperviscosity syndrome. Quantitative serum immunoglobulins showed elevated IgM. Discovering the MYD88 L265P in our patient’s biopsy sample was the first clue that his disease was not a routine CNS lymphoma. Bone marrow biopsy showed LPL suggesting WM. The patient underwent 3 plasma exchange treatments in the department of hematology followed by chemotherapy (cyclophosphamide for injection, bortezomib for injection), and the patient’s condition improved after treatment. However, we are unable to confirm the diagnosis due to lack of evidence from brain or meningeal biopsies and cerebrospinal fluid (CSF) immunophenotyping and genetic sequencing. In the next step, we will continue to follow up patients, hoping to improve brain magnetic resonance enhancement, CSF immunophenotyping, and gene sequencing.

Although rare, we may underdiagnose BNS. Its nonspecific neurological symptoms may resemble hyperviscosity syndrome and peripheral neuropathy in traditional WM. However, new, persistent, and asymmetric symptoms are atypical of WM alone and should raise suspicion for BNS. Clinicians must remain vigilant, as BNS may be the only sign of WM progression in a patient well-controlled on treatment. Furthermore, not only anemia, but up to one-third of cases of BNS cases can present as the initial manifestation of WM. Radiological features and the MYD88 L265P mutation are important clues pointing toward BNS, but definitive diagnosis requires CNS biopsy or CSF analysis. There remain no gold standard treatment rules for BNS. Because of its rarity, a randomized controlled trial is likely not feasible. Therefore, a high-quality meta-analysis of existing reports will be valuable to determine the optimal treatment.

## Author Contributions

Substantial contribution to the conception of the work: X.Y., C.Y.. Acquisition, analysis, and interpretation of the data: X.Y., Z.C.. Drafting: Z.C., X.Y.. Critical revision for important intellectual content: C.Y., G.W.. Agreement to be accountable for all aspects of the work: Z.C. on behalf of all authors. All authors read and approved the final manuscript.

**Conceptualization:** Xiaoqian Yang, ZongLei Chong.

**Data curation:** Chunxia Yan, Congcong Ma, Guifang Wang, Xiaoqian Yang, ZongLei Chong.

**Formal analysis:** Xiaoqian Yang, ZongLei Chong.

**Funding acquisition:** Chunxia Yan, Guifang Wang.

**Investigation:** Congcong Ma, Xiaoqian Yang, ZongLei Chong.

**Methodology:** Xiaoqian Yang, ZongLei Chong.

**Project administration:** Chunxia Yan, Guifang Wang, Xiaoqian Yang, ZongLei Chong.

**Resources:** Chunxia Yan, Guifang Wang, Xiaoqian Yang, ZongLei Chong.

**Software:** Chunxia Yan, Guifang Wang, Xiaoqian Yang.

**Supervision:** Xiaoqian Yang, ZongLei Chong.

**Validation:** Xiaoqian Yang, ZongLei Chong.

**Visualization:** Xiaoqian Yang, ZongLei Chong.

**Writing – original draft:** ZongLei Chong.

**Writing – review &amp; editing:** ZongLei Chong.
